# Automated detection of broiler vocalizations a machine learning approach for broiler chicken vocalization monitoring

**DOI:** 10.1016/j.psj.2025.104962

**Published:** 2025-03-04

**Authors:** Patricia de Carvalho Soster, Tomasz Grzywalski, Yuanbo Hou, Pieter Thomas, Annelike Dedeurwaerder, Maarten De Gussem, Frank Tuyttens, Paul Devos, Dick Botteldooren, Gunther Antonissen

**Affiliations:** a Department of Pathobiology, Pharmacology and Zoological Medicine, Faculty of Veterinary Medicine, Ghent University, 9820 Merelbeke-Melle, Belgium; b Poulpharm Bvba, Izegem, Belgium; c Department of Information Technology, Ghent University, 9052 Gent, Belgium; d Vetworks Bvba, Aalter, Belgium; e Flanders Research Institute for Agriculture, Fisheries, and Food (ILVO), 9090 Merelbeke-Melle, Belgium; f Department of Veterinary and Biosciences, Faculty of Veterinary Medicine, Ghent University, 9820 Merelbeke-Melle, Belgium

**Keywords:** Multi-branch neural networks, Transfer learning, Sound event detection, Broiler vocalization, Precision livestock farming

## Abstract

The poultry industry relies on highly efficient production systems. For sustainable food production, where maintaining broiler welfare is crucial, it is essential to have robust data collection systems and automated methods for assessing broiler health and welfare. This paper presents the development and implementation of an acoustic system designed to detect and differentiate between four distinct vocalizations of broiler chickens—pleasure notes, distress calls, short peeps, and warbles—while filtering out background noise and other vocalizations. The vocalization detector is designed as a convolutional neural network with 11 two-dimensional convolutional layers and one one-dimensional convolutional layer. For training, a manually labeled vocalization library was built (>2k samples, with a total duration of 190 minutes), based on a large set of continuous audio recordings of ten male Ross 308 broiler chicks aged from 1 to 36 days. An extension with a subset of the AudioSet dataset was made to include background sounds. With this library, an overall balanced accuracy of 91.1 % was achieved by the neural network-based recognizer.

## Introduction

The increasing demand for animal protein worldwide, driven by the high population growth rate, challenges the supply chain to be more efficient (FAO, 2019). Considering that broiler production presents a small profit margin, improvement in performance by reducing production cost is needed. Increasing pressure on the poultry industry to enhance productivity has led to larger flock sizes, making it increasingly difficult for producers to reliably assess animal behavior and welfare, as reviewed by Kiani (2022). In this context, there is a need for an efficient way to enhance production to meet food demand while simultaneously improving production control.

Precision livestock farming (PLF) is the use of a set of advanced technologies that can optimize animal performance and health by monitoring animals in real-time continuously and automatically (Berckmans, 2017), making it possible to collect information quickly to assess animal conditions, as reviewed by Banhazi, et al (2012). The application of audio analysis and more specifically the use of an acoustic vocalization detector could be part of such a management tool to assess animal health and welfare.

Broiler vocalization has been the subject of investigation in numerous studies (Aydin and Berckmans, 2016; Cuan et al., 2020; Rizwan et al., 2016). Broilers use vocalizations to express their internal and external states (Fontana et al., 2015b), using it to communicate and to express their needs (MacGrath et al., 2017; de Moura et al, 2008). Literature distinguishes four main types of vocalizations in broiler chicks in the first week of age based on the distribution of energy (0.001 to 2 dB), frequency (2 to 6.5 kHz), and duration (23 to 900 msec) (Marx et al., 2001). Distress calls, characterized by repetitive and high-energy vocalizations, are associated with stress situations (Marx et al., 2001). These calls might serve as powerful indicators of heightened emotional states or physical discomfort within individuals or flocks, conveying a need for attention or response. The frequency and intensity of distress calls vary depending on the nature and intensity of perceived threats or stressors (Neethirajan, 2023).

Short peeps are characterized by descending frequency, low energy, and a short range of duration (Marx et al., 2001). They have been observed to correlate with the level of activity exhibited by the chickens (Fontana et al, 2015b). Warbles, on the other hand, consist of repetitive bow-type elements with ascending or descending frequency and low energy (Marx et al., 2001), potentially related to somnolence. Pleasure notes, characterized by ascending frequency, low energy that tends to swing upward in pitch and a short range of duration (Marx et al., 2001), possibly related to positive welfare.

Historically, many attempts have been made to understand broiler vocalizations. Sound analysis for call detection has been explored as a potential tool for identifying various conditions, including environmental challenges such as heat and cold stress (Lee et al., 2015; Mortola, 2019), emotional states such as frustration (Zimmerman et al., 2000; Zimmerman et al., 2003), behavioral issues such as feather pecking (Bright, 2008), and performance-related factors such as feeding behavior (Aydin et al., 2015; Aydin & Berckmans, 2016).

Recently, automated methods have been tested. To identify real-time warnings of emerging welfare concerns, Herborn et al. (2020) made audio recordings of the first four days of 12 broilers. Distress calls were then identified based on spectral entropy. Jakovljević et al. (2019) tested support vector machines (SVMs, a maximum margin classifier that uses a hyperplane to separate classes in data) to detect stress in broilers through their sounds. A combination of MFCC signal representation and SVM classifier was also used by Lin and Lin (2015) to recognize chicken sounds. Similarly, Cheng and Zhong (2015) used a combination of MFCC, linear predictive coding (LPC) and power-normalized cepstral coefficients (PNCC) coupled with SVM to classify broiler vocalizations. In contrast, Sun et al. (2021) combined MFCC with kNN (k-Nearest Neighbors) and Random Forest Classifier to detect chicken sounds. A slightly different approach was proposed by Mao et al. (2022), who developed a lightweight version of the popular VGG11 neural network and trained it on log-mel spectrograms to perform binary classification that differentiated broiler distress calls from normal broiler house sounds.

The challenge of detecting broiler chickens' vocalizations includes dealing with many interfering noises commonly found in poultry houses, such as those from ventilation, heating, and feeding systems, as well as normal behaviors like wing flapping and dustbathing. (Yang et al., 2021; Jakovljević et al., 2019). Lin and Lin (2015) addressed the noise problem by proposing Anti-noise MFCC (AMFCC). The AMFCC is constructed by using a noise estimation algorithm to estimate the noise power spectrum of the chicken sounds in the noise environment followed by a multi-band spectral subtraction to achieve the clean sound spectrum. In a similar fashion, Cheng and Zhong (2015) first used a multi-band spectral subtraction method to reduce noise, then constructed chicken vocalization signals via sparse representation using the orthogonal matching pursuit algorithm to preserve the most important major structures with original signs. Sun et al. (2021) tested five signal filtering methods, namely Basic Spectral Subtraction, Improved Spectral Subtraction based on multi-taper spectrum estimation, Wiener filtering, thirty-atom Sparse Decomposition and fifty-atom Sparse Decomposition and evaluated their impact on the signal quality and the ability of machine learning classifiers to recognize four types of broiler sounds (crow, cough, purr and wing flapping).

The subject literature reveals that the in-situ broiler vocalization detection problem for application in realistic environments is still challenging and is typically limited to the detection of distress calls. In our study, we aimed at building on the previous advancements and creating a comprehensive solution that would fill the existing gaps. The main contributions of this work are:

• A database of broiler vocalizations was created, spanning the whole broiler life (1 to 36 days), to train and validate a broiler vocalization detector. This database includes currently known types of broiler vocalizations (distress calls, short peeps, warbles, pleasure notes) but also contains other sounds that could be explored later to distinguish new types of vocalizations.
• An advanced but well-established noise suppression algorithm (spectral gating, Sainburg et al, 2020) was employed to deal with background noise.
• And finally, the most advanced signal processing classifiers currently available, namely deep convolutional neural networks, were further developed and optimized, allowing accurate sound detection and identification in real-life environments. Focus was not only to detect distress calls, but also other known types of vocalizations.

## Material and methods

The study was carried out under the general certification number LA1400564, which permits Poulpharm to conduct animal trials. As stipulated by the Royal Decree of May 29, 2013, regarding the protection of animals used in scientific research (published on July 4, 2013), Ethics Committee approval was not required for this trial since the procedures involved did not result in significant pain or distress beyond what is normally expected from minor interventions.

This section describes the details of the development of a robust broiler vocalization detector. First, a database of broiler vocalizations was constructed by audio stream acquisition, sample extraction, and sample labeling. Next, the architecture of the detector is described.

### Broiler vocalization database

The construction of a broiler vocalization database consists of three key stages: (1) acquiring audio samples from a broiler pen, isolated to the greatest extent possible from external influences, (2) extracting audio samples containing probable broiler vocalizations utilizing publicly available neural network model for audio event detection (Hou et al. (2022), Kong et al. (2020)), and (3) manually categorizing broiler sounds into five predefined classes, which encompass four types of broiler vocalizations (pleasure notes, distress calls, short peeps and warbles) and other types of broiler vocalizations. The broiler vocalization database is created on a week-by-week basis from 1 to 36 days old to account for the gradual drop in the average peak frequency of vocalizations as broilers age and increase weight (Fontana et al., 2015a).

**Fig. 1:**
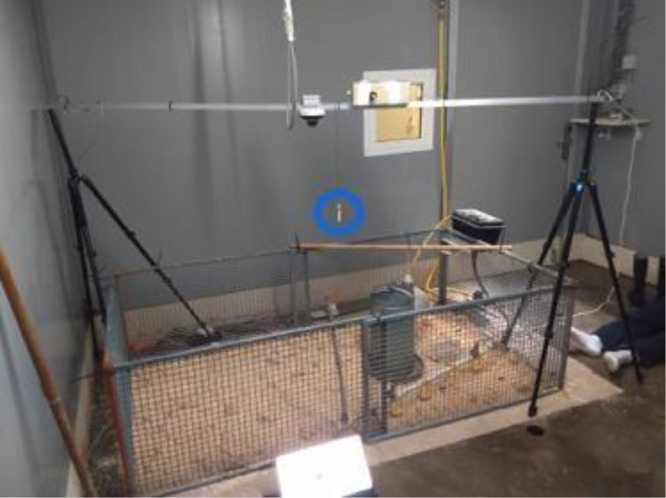
Broiler vocalization audio acquisition setup at Poulpharm (Izegem, Belgium). The position of the microphone (indicated with a blue circle) is central in the pen (1.1m x 2.1m), 90 cm above ground floor.

***Audio stream acquisition.*** Ten one-day-old male Ross 308 chicks, active and alert, with bright eyes, healthy legs, and a well-healed navel, free from deformities, weakness, or infection, were visually assessed following commercial hatchery standards (Aviagen, 2018). The birds were housed together in a single pen measuring 1.1m x 2.1m located in an isolated room at Poulpharm BVBA in Izegem, Belgium (Fig. 1).

This room was acoustically separated from other broilers and the external environment. The chicks were fed commercial pellet diets, consisting of starter feed from 1 to 14 days old and grower feed from 15 to 36 days old. Feed and water were provided *ad libitum* throughout the experiment. The pen was lined with wood shavings and equipped with a tube feeder and a bell drinker. The temperature on day 1 was 34 °C on average and gradually decreased by 1 °C every 2 days until 22 °C was reached, ensuring optimal comfort levels throughout the duration of the study. During the first week, chicks experienced a 1-hour dark period, which was extended to 6 hours thereafter. The lighting schedule consisted of lights turning on at midnight, off at 2 PM, on again at 6 PM, and off at 8 PM (Aviagen, 2019). Daily monitoring was conducted to observe any signs of illness or mortality. No deaths occurred in this study. General health status, body weight, mortality and the estimated cause of death were recorded.

A low-cost sensor node with a Knowles FG-23329 microphone (Van Renterghem et al., 2010) was positioned at a height of 90 cm in the center of the pen and continuously recorded chick vocalizations at a 48 kHz sampling rate. The microphone height was chosen as a trade-off between achieving uniform recording gain across the entire pen area (1.10 m × 2.10 m) and optimizing the signal-to-noise ratio by reducing interference from background noise and reflections from the pen walls. The selected 48 kHz sampling rate, based on previous studies (Huang et al., 2019; Liu et al., 2020; Lv et al., 2023), is sufficient to accurately capture the frequency range of chick vocalizations.

***Sample extraction.*** Possible interesting broiler vocalizations for the database were identified using the pure-convolution-based pre-trained audio neural network (PANN) to tag the recorded audio stream (Hou et al., 2022; Kong et al., 2020). The PANN model, featuring over 80M parameters, was pre-trained on AudioSet (Gemmeke et al., 2017), which is a large-scale database of audio sound events containing over 2M recordings, with 527 classes of audio events organized in a tree-like structure called ontology.

**Fig. 2:**
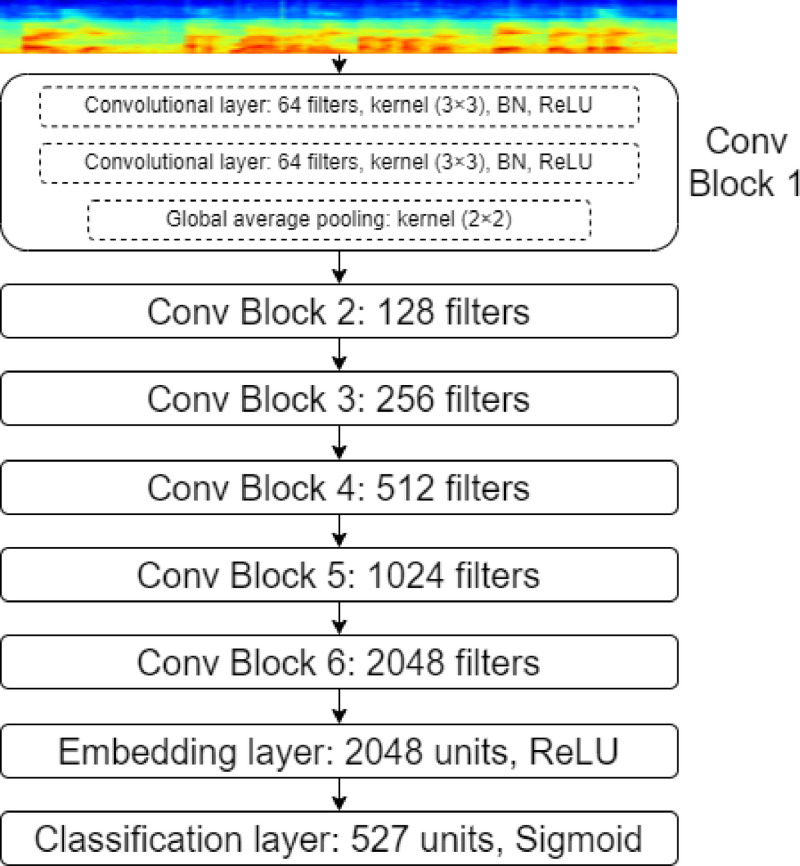
An overview of the PANN model architecture.

The AudioSet ontology was constructed specifically to cover most of the sound event types that people encounter in their daily lives. In terms of neural network architecture, the PANN model is composed of six convolutional blocks. Each convolutional block contains two convolutional layers, each using a kernel size of (3 × 3) followed by ReLU (rectified linear unit) non-linearity. After each convolution a batch normalization is used to stabilize and accelerate the training. Convolutional blocks are followed by a linear dense layer further followed by an output dense layer with a sigmoid activation function. The output layer predicts probability scores for the 527 classes of audio events. On input, the PANN model accepts a log-mel spectrogram representation of the input signal with 64 mel filter bank bins and 10 ms hop length. Please refer to Fig. 2 for an overview of the PANN model architecture.

A specific set of labels from the AudioSet ontology was selected to detect potential chicken vocalizations, including relevant terms such as 'Fowl,' 'Chicken, rooster,' 'Bird,' and 'Bird vocalization, bird call, bird song'. If the likelihood of these labels exceeded a threshold of 0.2 within any given second, that time segment was marked for extraction. Sound samples were then clipped from the audio stream, capturing one second before and after the identified interval. Overlapping samples were consolidated, and only those with the likelihood of at least 0.3 were retained, further refining the dataset. The resulting samples ranged from 3 to 45 s in length, each containing one or more potential vocalizations.

The sample extraction process was optimized to capture all potential broiler vocalizations, with false positives being identified during manual labeling. However, occasional false negatives, if present, were not considered critical. The aim of the sound extraction and subsequent manual labeling was to build a comprehensive database of broiler vocalizations, ensuring sufficient examples of each vocalization type across all weeks of the rearing period. Therefore, complete labeling of the acquired audio stream was not necessary.

***Sample labeling.*** In the subsequent phase, samples were manually labeled by a trained expert. The expert was trained using a standardized protocol, including a review of scientific literature, particularly Marx (2001), to understand the acoustic characteristics of broiler vocalizations. Labeling was conducted through auditory assessment and visual spectrogram analysis. To ensure consistency and eliminate inter-rater variability, all labeling was performed by a single expert following the standardized criteria. Intra-observer reliability was not formally tested. The samples were reviewed multiple times in search of examples of each type of vocalization from each week of the broiler rearing period. Our initial objective was to find at least 50 samples of each type of vocalization each week. In cases where enough samples were found, further review was ceased. In cases where not enough samples were found, review continued until all samples from a given period were reviewed. From any given week, samples were reviewed in a random order to ensure that no bias was introduced.

**Fig. 3:**
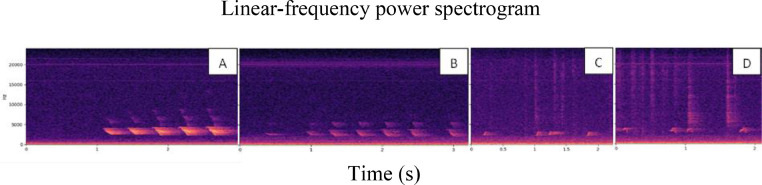
Example recordings from our database, presented as linear-frequency power spectrograms, including: (A) distress calls—repetitive, high-energy vocalizations; (B) short peeps—characterized by descending frequency, low energy, and short duration; (C) pleasure notes—featuring ascending frequency, low energy, an upward-swinging pitch, and short duration; and (D) warbles—repetitive bow-shaped elements with ascending or descending frequency and low energy.

The samples were categorized into four specific types: distress calls - repetitive, high-energy vocalizations; short peeps - characterized by descending frequency, low energy, and short duration; pleasure notes - featuring ascending frequency, low energy, an upward-swinging pitch, and short duration; and warbles - repetitive bow-shaped elements with ascending or descending frequency and low energy (Fig. 3). If none of these vocalizations were present, the sample was labeled as 'other sound'. Samples containing two or more types of vocalizations were labeled as a 'combination of sounds’ and not included in the database (however, they were analyzed and discussed separately in the Results section of this manuscript). The vocalizations were classified weekly (from week 1 to 5), allowing for accurate categorization of each vocalization according to its type and respecting the week age of the chicken to be able to account for the variability that may occur in vocalizations when broilers age.

### Architecture of the recognizer

This section describes the design of the neural network-based broiler vocalization detector.

***Neural network architecture.*** To achieve optimal performance, both in terms of accuracy and computational complexity, we proposed a custom neural network architecture designed specifically for the task of broiler chicken vocalization detection. The proposed neural network takes as input a time-frequency representation of the audio signal, specifically in the form of a log-mel spectrogram (Hou et al., 2023). After resampling the input signal to 16 kHz, it is transformed into a spectrogram using a 512-point Short-Time Fourier Transform (STFT) with a window size matching the STFT, a 160-sample (10 ms) hop length, and a Hann window. The STFT is then filtered with 64 mel filters covering a frequency range from 50 Hz to 8 kHz. The resulting mel spectrogram is converted to a decibel scale and linearly scaled to fit within the range of (-1, 1).

The neural network is designed as a fully convolutional model, comprising 11 2D convolutional layers and one 1D convolutional layer. Each 2D convolutional layer employs ELU (exponential linear unit) activation and is followed by batch normalization to accelerate convergence. The final 1D convolutional layer has an effective stride of 24 spectrogram frames (240ms) and contains six neurons. The first five neurons, responsible for broiler vocalization classification, use SoftMax activation, enabling the model to identify the dominant vocalization type at any given moment. The model's performance in situations with simultaneous vocalizations is also discussed in the Results section. The sixth neuron, which uses linear activation, estimates the normalized age of the broiler. Training the recognizer with broiler age as an additional training target helps the model capture the relationship between vocalization pitch and chicken age, thereby enhancing its accuracy.

The model's effective receptive field along the time axis spans 194 spectrogram frames, equivalent to 1.94 seconds of audio. Since the convolutional layers are all valid convolutions (i.e., without padding), this is also the minimum signal length needed for a prediction. During both pre-training and fine-tuning the same spectrogram length was used. Architecture of the proposed convolutional neural network for broiler vocalization detection is presented in Table 1. The model contains a total of 1.2 million trainable parameters and can process 60 seconds of audio in one second on an Intel Core i7-6700 CPU. The model was implemented in Python 3.8 using the TensorFlow 2.5 library.

**Table 1:** Architecture of the proposed convolutional neural network for broiler vocalization detection. Each layer is specified by its type, number of filters, size of filters and their strides. The final column specifies spatial dimensions (time, frequency) of the layer's output when the neural network is fed with an input spectrogram of 194 time frames and 64 frequency bands.

Layer type	Number of filters	Kernel size	Strides	Layer output shape
Conv 2D	64	3, 3	1, 1	192, 62
Conv 2D	64	3, 3	1, 1	190, 60
Conv 2D	96	4, 4	2, 2	94, 29
Conv 2D	96	3, 3	1, 1	92, 27
Conv 2D	128	5, 3	3, 2	30, 13
Conv 2D	128	3, 3	1, 1	28, 11
Conv 2D	128	4, 3	2, 2	13, 5
Conv 2D	128	3, 3	1, 1	11, 3
Conv 2D	128	3, 3	1, 1	9, 1
Conv 2D	128	3, 1	2, 1	4, 1
Conv 2D	128	4, 1	1, 1	1, 1
Conv 1D	6	1	1	1

***Pre-training on AudioSet.*** Prior to training the neural network on broiler sounds, it was initially pre-trained using the AudioSet dataset (Gemmeke et al., 2017). This pre-training process involved a subset of 100,000 recordings from AudioSet, with a focus on bird and fowl sounds. Specifically, the subset included all available recordings labeled as 'Bird' or 'Fowl' along with their 20 sub-labels, totaling approximately 35,000 recordings. An additional 65,000 recordings were selected to uniformly represent the other 505 labels in the AudioSet database. During pre-training, the network's final 1D convolutional layer was temporarily replaced by a 527-neuron layer with sigmoid activation. The pre-training was carried out over 100 epochs using the Adam optimizer with a learning rate of 0.001 and Mean Squared Error loss. Training batches consisted of 32 samples. Throughout this process, the Mean Average Precision was monitored on a small (3 %) validation set, and the best model weights were saved.

***Broiler vocalizations pre-processing.*** Despite many efforts made, the database recordings were still polluted, to some extent, with background noises such as equipment noise (ventilation, feeders, heating) and human voices. To obtain a clean database, the recordings were filtered from background noise using a state-of-the-art non-stationary noise suppression algorithm (Sainburg et al., 2020). As a result, the recognizer was trained using noise-free recordings. To ensure similar-quality input during inference, the inference pipeline also needs to include a noise suppression step, preferably realized using the same algorithm.

***Fine-tuning for broiler vocalization detection.*** Next, the pre-trained model was fine-tuned to specifically detect broiler vocalizations using a broiler vocalization database. The fine-tuning process was conducted in two phases. In the first phase, only the output 1D convolutional layer was trained, while the weights of the 2D convolutional layers were kept unchanged.

**Fig. 4:**
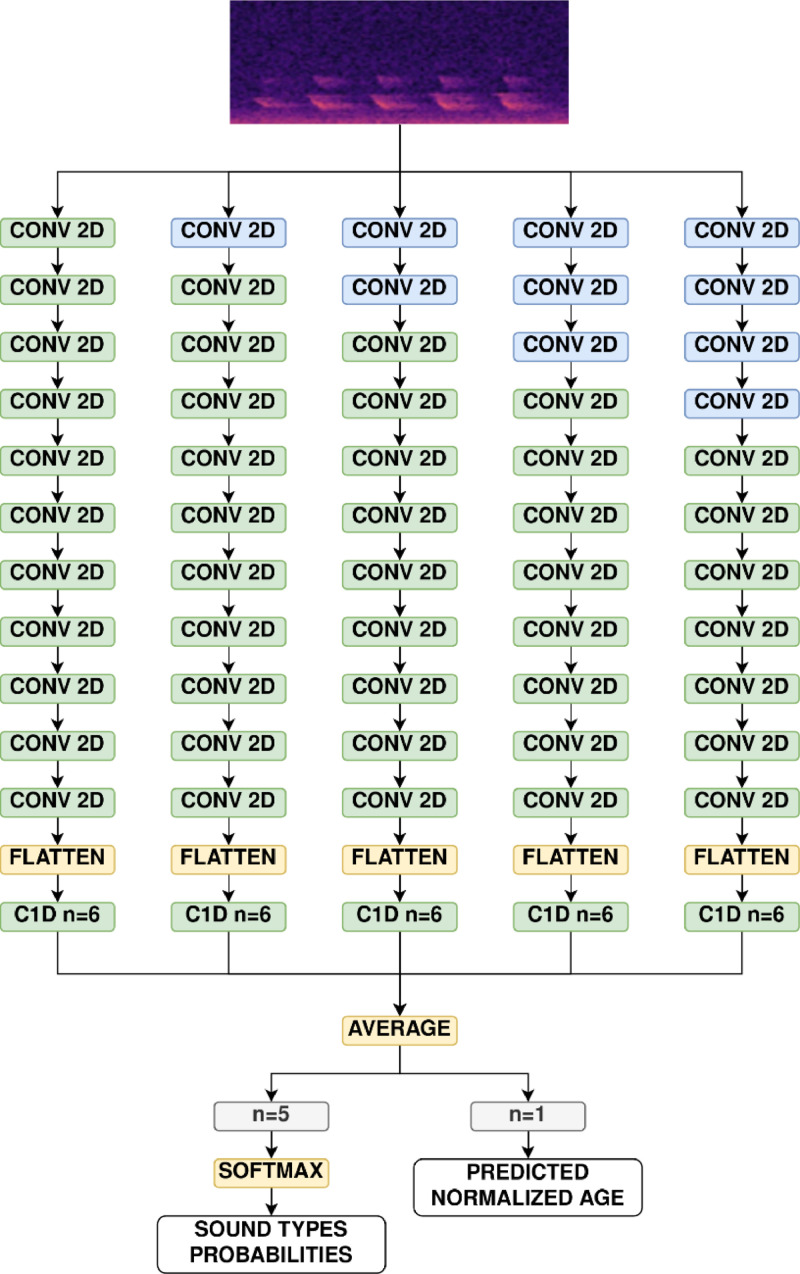
Branched structure of the broiler vocalization recognizer. Blue color indicates frozen weights during final fine-tuning.

In the second phase, five copies of the model, along with their weights, were created and combined into a single meta-model consisting of five branches (Fig. 4). In each branch a different number of initial 2D convolutional layers had their weights fixed (frozen) during the second phase of fine-tuning. Specifically, in the first branch, no weights were frozen; in the second branch, the weights of the first convolutional layer were frozen; in the third branch, the first two convolutional layers were frozen, and so on.

In both phases training was carried out for 50 epochs, with a batch size of 16, using the Adam optimizer with a learning rate of 0.001. The training data was balanced between sound classes using over-sampling of underrepresented classes. The loss function was a weighted combination of broiler vocalization classification loss (cross-entropy) and broiler age estimation loss (mean squared error), with the latter assigned a weight of 0.5 to reflect its relatively lower importance. Throughout the training, the key metric monitored was the balanced accuracy (BACC) of broiler vocalization classification on a held-out validation set, which included 25 randomly selected recordings for each vocalization type. This metric determined when to save the final model weights snapshot.

***Final design: inclusion of background noises.*** The recognizer described thus far can differentiate between different types of broiler vocalizations only, but it cannot detect when broilers are not vocalizing, which is a necessary condition when deployment in realistic environments is targeted. To facilitate continuous broiler monitoring without dependency on external systems, such as a PANN pre-analysis, the recognizer has been extended with an additional output to indicate absence of vocalizations. The positive training examples for this 7^th^ output neuron have been gathered from AudioSet (Gemmeke et al., 2017). A sample of 8k recordings has been used that uniformly represents all the AudioSet ontology classes except all Animal labels, which were excluded since they might contain actual broiler vocalizations. The selected set covers a wide range of sounds including those expected in the stable, e.g. human activity, ventilation noise and other mechanical sounds.

## Results and discussion

### Broiler vocalization labeling

In total, the constructed broiler vocalization database contains 2559 audio recordings with a total duration of 190 min. These recordings were manually labeled as distress calls (n = 486), pleasure notes (n = 803), short peeps (n = 622), warbles (n = 306) and other broiler vocalizations (n = 342). This set of labels covers currently well described types of broiler chicken vocalizations (Marx et al., 2001), while the final category could be used in the future to discover new concrete vocalization types. Detailed structure of the database is presented in Table 2. Additional to the recordings included in the database, 4583 recordings of a cumulative duration of 316 min were labeled as “combination of sounds” and not included in the database. Examples of the four types of vocalizations taken from the database are shown in Fig. 3.

**Table 2:** Composition of the broiler vocalizations database by week of broiler chickens' age, including the number of audio samples and total duration. In total, the database includes 2559 recordings of broiler vocalizations of a total duration of 3 hours and 10 minutes.

Sound category	Week 1	Week 2	Week 3	Week 4	Week 5
Distress calls	188 (23 min.)	48 (3.7 min.)	136 (15.0 min.)	55 (4.8 min.)	59 (4.7 min.)
Pleasure notes	685 (53.1 min.)	67 (4.2 min.)	51 (3.1 min.)	0	0
Short peeps	51 (3.6 min.)	212 (16.0 min.)	154 (13.3 min.)	151 (9.9 min.)	54 (3.6 min.)
Warbles	24 (1.1 min.)	85 (3.6 min.)	96 (4.8 min.)	49 (2.1 min.)	52 (2.1 min.)
Other sounds	83 (5.2 min.)	22 (1.4 min.)	59 (3.1 min.)	128 (7.0 min.)	50 (2.6 min.)

It is important to understand that our primary aim was not to achieve a comprehensive classification of all recorded audio streams, but rather to construct a database of high-quality vocalization samples for the development of a recognizer. To ensure the quality of our database, stringent acceptance criteria were set for both sample extraction (based on the PANN model) and manual labeling processes. The numbers presented in Table 2 only partially reflect the prevalence of different vocalization types across various ages of broilers. In instances where sufficient samples were obtained for a specific sound type at a particular age, further sampling was ceased. Conversely, additional effort was invested in locating scarce samples. This approach enabled us to curate a dataset that is more balanced than if samples were gathered proportionally.

It should be noted that, although in the first week a high number of pleasure notes was recorded, none could be detected in the last two weeks, raising questions about whether these calls cease to be produced. An alternative perspective could consider that the absence of pleasure notes in later weeks could be attributed to their low intensity in an environment where noise increases as broilers age, such as ventilation hum. Furthermore, it is plausible that this vocalization evolves over time, potentially leading to the decrease in numbers observed in subsequent weeks, as new forms of vocalization may emerge to indicate pleasure that are no longer recognized as the original category.

### Acoustic characteristics of broiler vocalizations

To better understand the differences between vocalization types and how they change as broilers age, we analyzed the acoustic features of audio recordings from our database. The results are presented in Table 3.

**Table 3:** Acoustical characteristics of four types of broiler vocalizations discretized in weeks. The vocalizations are described using four features: sound length expressed in seconds, maximal acoustic power expressed in decibels, fundamental frequency at the point of maximal energy expressed in Hz, and fundamental frequency trend – change of the fundamental frequency across the call's duration, expressed in Hz per 10 ms. For each vocalization type and week, two statistics are drawn: mean value and standard deviation.

Vocalization type	Week	Sound length [s]	Maximal acoustical power [dB]	Fundamental frequency [Hz]	Fundamental frequency trend [Hz per 10 ms]
Distress calls	1	0.19 ± 0.08	-43.4 ± 7.5	2994 ± 398	-48.9 ± 28.0
	2	0.20 ± 0.09	-51.8 ± 7.1	3005 ± 189	-66.3 ± 25.3
	3	0.18 ± 0.08	-46.3 ± 7.6	2786 ± 741	-45.3 ± 37.8
	4	0.22 ± 0.11	-48.9 ± 8.8	2238 ± 748	-20.6 ± 31.4
	5	0.20 ± 0.10	-45.5 ± 8.6	1617 ± 856	-9.1 ± 31.7
Pleasure notes	1	0.21 ± 0.11	-62.5 ± 2.8	2945 ± 388	30.6 ± 27.1
	2	0.22 ± 0.11	-61.6 ± 3.7	2687 ± 272	17.5 ± 26.3
	3	0.21 ± 0.10	-58.2 ± 3.5	2483 ± 437	8.4 ± 25.0
Short peeps	1	0.19 ± 0.07	-58.5 ± 5.2	2839 ± 441	-55.7 ± 39.3
	2	0.27 ± 0.12	-60.4 ± 3.7	2991 ± 399	-44.9 ± 32.3
	3	0.23 ± 0.11	-57.7 ± 6.0	2651 ± 698	-42.7 ± 36.5
	4	0.22 ± 0.14	-59.0 ± 4.4	2478 ± 579	-28.4 ± 34.3
	5	0.20 ± 0.12	-55.4 ± 5.8	2002 ± 792	-27.5 ± 29.9
Warbles	1	0.30 ± 0.15	-63.6 ± 2.3	3110 ± 302	27.8 ± 24.1
	2	0.35 ± 0.13	-60.5 ± 4.0	2854 ± 639	-5.7 ± 23.6
	3	0.30 ± 0.19	-60.5 ± 4.0	2771 ± 736	-19.0 ± 37.1
	4	0.29 ± 0.17	-59.9 ± 4.1	2481 ± 464	-9.3 ± 24.5
	5	0.37 ± 0.23	-55.8 ± 3.8	1197 ± 1382	-6.0 ± 9.9

To this end, we extracted individual calls from each audio sample in our database (containing one or more calls) using classical signal processing techniques, i.e. calculation of short-windowed power envelope followed by dynamic thresholding to identify cutting points. Next, from each call, we extracted four features: sound length expressed in seconds, maximal acoustic power expressed in decibels, fundamental frequency at the time of maximal energy expressed in Hz, and fundamental frequency trend, which summarizes the change in fundamental frequency across the call's duration and is expressed in Hz per 10 ms. We note that the last feature is particularly effective in describing the direction of change of the call's pitch (positive values for ascending or negative values for descending frequency) and the steepness of the change (higher absolute values are related to higher steepness). The fundamental frequency in each audio frame, used for obtaining the latter two features, was calculated using the probabilistic YIN (pYIN, Mauch et al., 2014) method. As a final step, we calculated the mean feature value and standard deviation separately for each vocalization type and week.

In general agreement with the findings of Fontana et al., 2015a, the fundamental frequency of calls decreased from approximately 3 kHz in the first week to 1.2 - 2.0 kHz (depending on the vocalization type) by week 5. Notably, different vocalization types exhibited similar fundamental frequencies throughout the rearing period, particularly at younger ages, suggesting that pitch is not a reliable discriminator of call type.

Further insights are provided by the fundamental frequency trend. In agreement with previous findings (Marx et al., 2001), distress call and short peeps exhibited a clearly descending frequency pattern, with the steepness of the descent decreasing as broilers aged. The only exception from this was observed in distress calls, where 2-week-old broilers had a steeper pitch descent (-66.3 Hz per 10 ms) than 1-week-old broilers (-48.9 Hz per 10 ms). In older broilers, the pitch descent of distress calls was significantly reduced, reaching only -9.1 Hz per 10 ms. In the same age group, short peeps maintained a relatively steep descent of -27.5 Hz per 10 ms.

In contrast, pleasure notes exhibited an ascending frequency pattern, with the steepness of the ascent decreasing as broilers aged. Warbles, previously characterized as bow-type elements, also reflected this pattern in our data. For weeks 2, 4 and 5, the parameter value remained close to 0, consistent with the expected bow-shaped frequency contour. However, in week 1, the value was relatively high (27.8 Hz per 10 ms), indicating that in this age group, on average, the ascending portion of the bow was more prominent. In contrast, in week 3, the parameter value became negative (-19.0 Hz per 10 ms), suggesting that at this stage, the descending portion of the bow might be more pronounced.

The acoustic power of the calls did not exhibit a clear pattern; however, by week 5, most calls were louder compared to earlier weeks. As expected, distress calls were by far the loudest vocalizations, while the other three call types showed similar acoustic levels with a slight tendency to become louder as the broiler aged.

Regarding call lengths, distress calls and pleasure notes remained stable at approximately 200 ms across all weeks. Short peeps had similar durations in weeks 1 and 5, but were slightly longer in weeks 2 to 4. Warbles were the longest vocalizations, with average durations ranging from 300 ms to 370 ms.

Despite the strong agreement of our findings with existing literature, it is important to note that the presented statistics exhibit relatively high standard deviations, indicating significant overlap between different vocalizations in these simple acoustic features. This highlights the inherent difficulty in distinguishing vocalization types and supports the use of deep neural networks for classification, as they can capture more nuanced patterns. Additionally, despite extensive efforts, some extracted calls still contained two or more calls emitted in quick succession, therefore the “sound length” statistic should be treated with some caution.

### Vocalization detector performance

Each configuration of the recognizer considered was tested by training the model ten times, each time excluding a different random test set from the training and validation data. In every instance, the test set consisted of 25 example recordings from each sound category. These test sets were utilized only once after training the respective model to assess its effectiveness in sound classification. The results from each of the ten experiment repetitions were aggregated and jointly summarized. Consequently, the reported results represent a combined test dataset of 1250 recordings for the model developed in section 2.2.4, and 1500 recordings for the model described in section 2.2.5.

Performance of the recognizer in classification of vocalizations is measured in terms of precision, recall, f1-score and accuracy (class-wise and balanced). Here, precision is the number of examples correctly identified as given sound type divided by the total number of examples identified as given sound type. Recall (sensitivity) is the fraction of examples of given sound that were correctly identified as given sound type. F1-score is the harmonic mean of precision and recall. Class-wise accuracy is the total number of examples correctly identified as given sound type or correctly identified as not being of given sound type divided by the total number of test examples. The global accuracy is the total number of correctly identified test examples divided by the total number of test examples. Because the test set used in this study was balanced, the global accuracy is equal to balanced accuracy (BACC). The recognizer's ability to correctly estimate broiler age is measured using mean absolute error (MAE).

When submitting the detector, developed in section 2.2.4 and constructed to distinguish between the four different types of vocalizations and ‘other’ broiler vocalization sounds, to the proposed evaluation method, an overall broiler vocalization classification balanced accuracy of 87.9 % is achieved. An accuracy of 97.7 %, 96.4 %, 92.3 %, 95.0 % was observed for distress calls, pleasure notes, short peeps, and warbles, respectively. Detailed results are shown in Table 4.

**Table 4:** Accuracy, precision, recall, and F1-Score of the broiler vocalization classifier. Precision is the number of examples correctly identified as given sound type divided by the total number of examples identified as given sound type. Recall (sensitivity) is the fraction of examples of given sound that were correctly identified as given sound type. F1-score is the harmonic mean of precision and recall. Class-wise accuracy is the total number of examples correctly identified as given sound type or correctly identified as not being of given sound type divided by the total number of test examples.

Sound category	Accuracy	Precision	Recall	F1-score
Distress calls	97.7%	91.7%	97.2%	94.4%
Pleasure notes	96.4%	93.6%	88.0%	90.7%
Short peeps	92.3%	78.7%	84.4%	81.5%
Warbles	95.0%	87.3%	87.6%	87.4%
Other sounds	94.5%	89.2%	82.4%	85.7%
Average	95.2%	88.1%	87.9%	87.9%

Table 5 illustrates how various components of the proposed solution impact the detector's performance. An ablation study was conducted by removing or disabling individual features of the solution wherever possible. However, isolating the effect of pre-training on the recognizer's performance proved challenging, as the multi-branch approach was specifically designed to enhance knowledge transfer from the pre-trained model. Eliminating pre-training from the branched model would be counterproductive, as it would result in freezing random weights within the branches. Therefore, the ablation study compares the performance of the multi-branch model with a single-branch model, both utilizing pre-trained weights. Additionally, the study separately examines the impact of pre-training on the performance of a single-branch model.

**Table 5:** Impact of various components of the proposed solution on the detector's performance (ablation study).

Age as extra training target	De- noising	Multi- branch	Pre- training	Broiler vocalization detection - BACC	Broiler age estimation - MAE [days]
Yes	Yes	Yes	Yes	87.9%	2.02
No	Yes	Yes	Yes	87.2%	-
Yes	No	Yes	Yes	80.8%	1.72
Yes	Yes	No	Yes	86.5%	2.76
Yes	Yes	No	No	84.2%	4.47

As shown, among the elements considered in the proposed recognizer's pipeline, the most significant improvement (7.1 percentage points) came from incorporating nonstationary noise suppression. Starting with pre-trained weights in a single-branch model resulted in a 2.3 percentage point increase in performance compared to training from random weights. Additionally, the use of multiple branches to more effectively leverage the pre-trained weights further enhanced broiler vocalization classification by an additional 1.4 percentage points. Altogether, the optimized transfer of knowledge improved balanced accuracy by 3.7 percentage points. The smallest improvement was observed when broiler age was added as an additional training target.

**Fig. 5:**
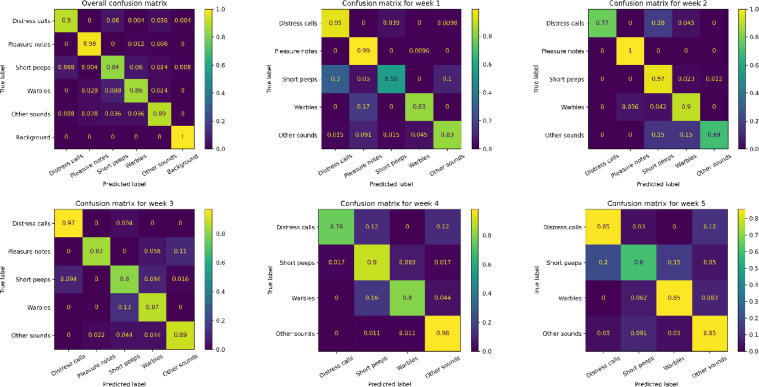
Overall (upper left) and per-week confusion matrixes of the broiler vocalization recognizer with an additional output class (“background”).

The vocalization recognizer, re-trained with an additional output (background noises), as described in Section 2.2.5, did not show degradation of accuracy in differentiation between different types of vocalizations and has shown excellent capability to detect when broilers were not vocalizing. In fact, the balanced accuracy in differentiation between vocalization types has increased from 87.9 % to 89.4 % in the upgraded model. When considering all output classes, the new recognizer's balanced accuracy is 91.1 %. Table 6 shows details about the performance of the upgraded version of the recognizer while Fig. 5 depicts the recognizer performance in the form of confusion matrixes (overall and on a week-by-week basis).

**Table 6:** Accuracy of the broiler vocalization recognizer with an additional output class (“background”), indicative of absence of broiler vocalizations. Numbers in brackets indicate change compared to the recognizer's performance without background class. Precision is the number of examples correctly identified as given sound type divided by the total number of examples identified as given sound type. Recall (sensitivity) is the fraction of examples of given sound type that were correctly identified as given sound type. F1-score is the harmonic mean of precision and recall. Class-wise accuracy is the total number of examples correctly identified as given sound type or correctly identified as not being of given sound type divided by the total number of test examples.

Sound category	Accuracy	Precision	Recall	F1-score
Distress calls	97.1% (-0.5)	92.2% (+0.5)	90.4% (-6.8)	91.3% (-3.1)
Pleasure notes	98.5% (+2.1)	93.2% (-0.4)	98.4% (+10.4)	95.7% (+5.0)
Short peeps	94.3% (+1.9)	82.3% (+3.6)	83.6% (-0.8)	82.9% (+1.4)
Warbles	95.8% (+0.8)	88.8% (+1.5)	85.6% (-2.0)	87.2% (-0.2)
Other sounds	96.7% (+2.2)	91.0% (+1.8)	88.8% (+6.4)	89.9% (+4.2)
Background	99.9%	99.2%	100%	99.6%
5-class average (no background)	96.5% (+1.3)	89.5% (+1.4)	89.4% (+1.4)	89.4% (+1.5)
6-class average (with background)	97.0%	91.1%	91.1%	91.1%

When analyzing the overall confusion matrix, the detector can almost perfectly distinguish when broilers are vocalizing. Background sounds have a recall of 100 %, while less than 1 % of broiler vocalizations are confused with background sounds. The overall recall of pleasure notes is highest (98 %), followed by distress calls (90 %). Distress calls and pleasure notes are never confused with each other. The recall of short peeps and warbles is somewhat lower, 84 % and 86 % respectively. These findings align with the analysis of acoustical features provided in Table 3. The high recall of pleasure notes can be attributed to their distinctive ascending pitch, which differentiates them from other vocalizations. Similarly, the high recall of distress calls may be linked to their strong acoustic power. However, some confusion arises with short peeps, which exhibit a similar ascending frequency pattern.

The analysis of week-by-week confusion matrices indicates a correlation between recognizer performance and the number of training samples available in the vocalization database (Table 2). For example, the performance of the detection of distress calls is the highest (recall above 90 %) in weeks 1 and 3, coinciding with the highest number of training samples for distress calls (188 and 136, respectively). In week 2 and 4 however, the recall of distress calls drops to about 76 %, and some are being confused with short peeps. This observation, combined with the analysis of acoustical features, suggests that even within the same category of vocalizations, sounds produced by broilers of different ages differ significantly. As a result, a sufficient number of examples needs to be provided for each week of the broiler age to ensure accurate detections during the whole life span of chickens.

A similar conclusion applies to short peeps, where recall is lowest in weeks 1 and 5 (55 % and 60 %, respectively), coinciding with the fewest samples in the database. This may suggest that short peeps produced by very young (week 1) and older (week 5) broilers have different acoustic properties compared to those at intermediate ages, although feature analysis does not provide clear evidence of this. However, since peeping behavior in young chicks is often linked to exploratory behaviors or mild distress (Fontana et al., 2015a), it is possible that the function or acoustic structure of these calls evolves as broilers mature.

Regarding pleasure notes, we see high recall values for week 1 and 2 (>99 %). In week 3 the recall drops to 83 % and 11 % of pleasure notes is confused with ‘other sounds’, which could indicate that broilers begin altering how they vocalize pleasure as they age, either due to physical maturation of the vocal apparatus or changes in behavioral context. No typical pleasure notes have been detected in weeks 4 and 5, hence these are omitted from the confusion matrices for the corresponding week, suggesting a potential disappearance or transformation of these calls later in life. This aligns with findings from Fontana et al. (2015a), which suggest that vocalization patterns shift over time.

For warbles, recall values remain relatively stable, fluctuating between 80 % and 90 % across all weeks. Most misclassifications occur with pleasure notes in week 1 and short peeps in weeks 3 and 4, which aligns with their acoustic characteristics. In week 1, warbles exhibit more ascending frequency patterns, making them similar to pleasure notes, while in week 3, they shift to a more descending pitch, resembling short peeps. These findings suggest that vocalizations evolve in spectral and temporal properties as broilers age, influencing recognizer performance.

Based on our experiments, we advise that 100 examples of a given vocalization type per week of broiler age should be sufficient to achieve high detection accuracy.

Considering previous efforts for automatic detecting broiler vocalizations, our proposed system bears some resemblance to Mao et al. (2022) which also used a deep and lightweight convolutional neural network (VGG16) that accepts as input a log-mel spectrogram. However, there the focus was on the identification of distress calls only (with a reported accuracy of 95 %), which limits its potential for application in broiler welfare monitoring. The advantage of our approach is that for the first time a model can detect four of the most frequent types of broilers vocalizations with high accuracy: we obtained a balanced accuracy for the four types of vocalizations of 91.1 %, with an accuracy in detecting distress calls of 97.1 % and the highest accuracy achieved for pleasure notes (98.5 %).

To evaluate the recognizer's performance when handling input signals containing a mix of broiler vocalizations, all sound samples that were manually labeled as 'combination of sounds' were processed using the trained model. The results showed that in most cases (∼86 %), the recognizer successfully identified one vocalization type with high confidence (probability > 80 %). This suggests that when faced with a combination of broiler vocalizations, the recognizer is generally capable of identifying the dominant one.

Given these promising results, we aim to further validate our solution and develop it into a commercially available product. The next steps include deploying the model on an edge device, for which a prototype has already been developed and successfully tested in a controlled environment. This will be followed by extensive validation in real farming conditions. The final critical step is integrating the detector into an electronic farm control system, requiring the definition of alarms, interfaces, and data transfer and storage protocols, all of which fall within standard engineering processes.

## Conclusions and future work

This work presented the development process and performance analysis of an automated broiler vocalization detector for four different types of broiler vocalizations. Our main contributions included (1) the creation of a broiler vocalization database, (2) designing and training a dedicated convolutional neural network to perform sound classification, and (3) making it self-sufficient, scalable and robust in preparation for large-scale deployment.

The vocalization database constructed in this work includes 2559 clean recordings of broiler vocalizations of a total duration of 190 minutes, representing the full broiler life span on a week-by-week basis. These recordings were manually labeled as distress calls (n = 486), pleasure notes (n = 803), short peeps (n = 622), warbles (n = 306) and other broiler vocalizations (n = 342). This set of labels covers all currently known types of broiler vocalizations while the final category can be used in the future to discover new vocalization types.

The vocalization detector is constructed as a deep convolutional neural network with eleven 2D and one 1D convolutional layers and a total of 1.2 million parameters and was trained with the vocalization database to perform sound classification based on the log-mel spectrogram of the input audio signal. To boost the robustness for deployment in realistic environments, the proposed solution relies on state-of-the-art noise suppression using spectral gating, which ensured an increase in accuracy of 7.1 percentage points (p.p.). To be able to distinguish when chickens are not vocalizing, the detector was retrained based on an extended version of the vocalization database with a selection of AudioSet recordings, representative of possible “background” sounds. The upgraded recognizer achieves a considerable overall balanced accuracy of 91.1 % with limited confusion and can process 60 seconds of audio stream in one second on a 6th generation Intel i7 processor. In terms of detection of distress calls, the recognizer achieves an accuracy of 97.1 %, which is an improvement of 2.1 percentage points as compared to the most advanced solution described thus far in the literature.

In its current design, simultaneous occurrences of multiple vocalization types cannot be detected, as only one type at a time is recognized with high probability (typically distress calls, when they occur). This limitation should be kept in mind when researching overall broiler behavior beyond farming context, but when deploying the technology in commercial farming settings, occurrence of distress calls is typically of main concern. To increase accuracy, however, multiple vocalization detectors could be deployed, each covering only a limited area of the broiler pen, allowing application in further research aimed at studying chicken behavior, welfare, or communication. Further research might focus on the deployment of the detector to investigate the typical vocalization behavior of broilers and the categorization and interpretation of the ‘other vocalization’ sounds. During the vocalization database construction, only little or no samples of positive welfare (warbles or pleasure notes) were found at older age. It is our assumption that broilers display signs of positive welfare differently when ageing. However, at this point no meaning or nuances within the ‘other sounds’ category have been investigated.

## Declaration of competing interest

Maarten De Gussen, Frank Tuyttens, Paul Devos, Dick Botteldooren and Gunther Antonissen reports financial support was provided by Flanders Innovation & Entrepreneurship. Maarten De Gussen, Frank Tuyttens, Paul Devos, Dick Botteldooren and Gunther Antonissen reports financial support was provided by imec ICON project WISH. If there are other authors, they declare that they have no known competing financial interests or personal relationships that could have appeared to influence the work reported in this paper.

## Appendix Supplementary materials

Image, application 1

Image, application 2

Image, application 3

Image, application 4

Image, application 5

Image, application 6

Image, application 7

Image, application 8

Image, application 9

Image, application 10

Image, application 11

Image, application 12

Image, application 13

Image, application 14

Image, application 15

Image, application 16

Image, application 17

Image, application 18

Image, application 19

Image, application 20

Image, application 21

Image, application 22

Image, application 23

Image, application 24

Image, application 25

Image, application 26

Image, application 27

Image, application 28

Image, application 29

Image, application 30

Image, application 31

Image, application 32

Image, application 33

Image, application 34

Image, application 35

Image, application 36

Image, application 37

Image, application 38

Image, application 39

Image, application 40

Image, application 41

Image, application 42

Image, application 43

Image, application 44

Image, application 45

Image, application 46

Image, application 47

Image, application 48

Image, application 49

Image, application 50

Image, application 51

Image, application 52

Image, application 53

Image, application 54
